# Effects of blood flow restriction training in adult upper-limb musculoskeletal rehabilitation: A systematic review

**DOI:** 10.1097/MD.0000000000051205

**Published:** 2026-10-16

**Authors:** Lama Saif Khan, Abdulelah K. Alqawlaq, Majd Hussain Almech, Abdulaziz Khalid Alaryani, Yousef I. Alhusaini, Adnan Alaa Sultan, Fouad Turki Alnajjar, Abdullah Mohammed Falqi, Wahib Shehab Alshaibani, Mohamed Ibrahim Hassan, Ramy Samargandi, Maan Jamjoom

**Affiliations:** a Emergency Medical Services, Medical Fakeeh, Jeddah, Saudi Arabia; b General Medicine Practice Program, Batterjee Medical College, Jeddah, Saudi Arabia; c Critical Care in Emergency Medical Service, Johns Hopkins Aramco Healthcare, Abqaiq, Saudi Arabia; d College of Medicine, King Saud bin Abdulaziz University for Health Sciences, Jeddah, Saudi Arabia; e Department of Orthopedic Surgery, International Medical Center, Jeddah, Saudi Arabia; f Department of Orthopedic Surgery, College of Medicine, University of Jeddah, Jeddah, Saudi Arabia; g Department of Emergency Medicine, Ministry of the National Guard Health Affairs, Jeddah, Saudi Arabia; h King Abdullah International Medical Research Center, Jeddah, Saudi Arabia.

**Keywords:** blood flow restriction training, distal radius fracture, hand osteoarthritis, Lateral elbow tendinopathy, postoperative rehabilitation, rotator cuff tendinopathy, upper-limb musculoskeletal rehabilitation

## Abstract

**Background:**

Blood flow restriction training (BFRT) has been proposed as a low-load rehabilitation strategy for symptomatic and postoperative upper-limb musculoskeletal conditions. This review evaluated the clinical effects, tolerability, and prescription characteristics of BFRT in adult upper-limb musculoskeletal rehabilitation.

**Methods:**

This systematic review was registered in International Prospective Register of Systematic Reviews (CRD420261299726) and reported with reference to Preferred Reporting Items for Systematic Reviews and Meta-Analyses 2020. CENTRAL/Cochrane Library, Embase via Ovid, MEDLINE/PubMed, Science Citation Index/Web of Science, and Scopus were searched without a start-date restriction before February 18, 2026; backward reference-list/citation checking was also used. Randomized trials, case series, and case reports were eligible. Findings were synthesized narratively because of heterogeneity, and studies were appraised using risk of bias 2 or design-appropriate Joanna Briggs Institute checklists.

**Results:**

Twenty reports were included: 9 parallel-group randomized trials, 1 randomized crossover trial, 2 case series, and 8 case-report publications. Across the reports, 340 participants were enrolled or randomized and 326 contributed to the analyzed/treated clinical samples; 158 were men (48.5%) and 168 were women (51.5%). In a sham-controlled lateral elbow tendinopathy trial, BFRT resulted in a significantly greater reduction in 12-week pain (between-group difference −1.54, 95% confidence interval [CI] −2.89 to −0.18; *P* = .026) and patient-rated tennis elbow evaluation (PRTEE) (−15.23, 95% CI −23.57 to −6.90; *P* < .001). In patients with chronic lateral epicondylitis, PRTEE reduction (−24.20 ± 4.02 vs −15.60 ± 3.71; *P* = .001) and quick disabilities of the arm, shoulder and hand reduction (−12.83 ± 4.97 vs −7.51 ± 4.72; *P* = .025) were significantly greater with BFRT plus conventional therapy, whereas between-group pain and strength changes were not significant. In nonoperative distal-radius-fracture rehabilitation, mean activity-pain reduction was 4.0 versus 2.3 points (*P* = .03). In rotator-cuff tendinopathy, BFRT resulted in significantly greater biceps thickness (*P* = .002) and internal-rotation strength (*P* = .040). Conversely, a pragmatic elbow trial found no significant between-group difference in 12-month PRTEE (adjusted contrast + 4.32, 95% CI −9.17 to 17.81; *P* = .53).

**Conclusion:**

BFRT may be considered a low-load adjunct in adult upper-limb musculoskeletal rehabilitation settings, but it is not consistently superior to active rehabilitation for pain, strength, or function. Confidence remains limited by small samples, heterogeneous prescriptions and co-interventions, uncontrolled designs, and incomplete adverse-event ascertainment.

## 1. Introduction

Upper-limb musculoskeletal disorders, including lateral elbow tendinopathy (LET), rotator-cuff-related tendon disorders, fractures, and postoperative conditions, can cause persistent pain, weakness, activity limitation, and work disability. ^[1–4]^ Tendinopathy is a major component of this clinical burden.^[1–4]^ In a population-based study, the age- and sex-adjusted annual incidence of lateral elbow tendinosis decreased from 4.5 per 1000 people in 2000 to 2.4 per 1000 in 2012, but recurrence within 2 years remained 8.5%.^[2]^ A related population-based analysis found substantial healthcare utilization and a mean direct medical cost of $660 per patient during the 1st year after diagnosis in 2013 US dollars, with higher costs among those requiring surgery.^[3]^ Tendinopathy more broadly also imposes socioeconomic costs through persistent symptoms, healthcare use, and work-productivity loss.^[4]^

Progressive resistance exercise is central to conservative tendinopathy rehabilitation because appropriately dosed loading can reduce pain and improve function.^[1,5]^ However, heavy mechanical loading may be poorly tolerated when symptoms are highly irritable or when postoperative restrictions temporarily constrain tissue loading. Blood flow restriction training (BFRT) combines low-load exercise with external pressure applied proximally to the exercising limb and is commonly prescribed at approximately 20% to 40% of 1-repetition maximum (1RM), with cuff pressure individualized to limb occlusion pressure (LOP) or delivered using fixed-pressure approaches.^[6–10]^ Original studies and previous reviews assessing the efficacy of BFRT in upper-limb rehabilitation have reported reductions in pain and disability and improvements in selected strength and muscle-morphology outcomes, although these effects have not been consistent across conditions, outcomes, or comparators.^[11–19]^ Current evidence also does not establish a single optimal BFRT load or cuff-pressure prescription for upper-limb rehabilitation.

Proposed mechanisms through which BFRT may produce these clinical and muscular adaptations include increased metabolic stress, earlier recruitment of higher-threshold motor units, cell swelling, and anabolic signaling.^[8–10]^ These mechanisms provide biological plausibility for strength and hypertrophy adaptations during low-load exercise, but they do not establish clinical superiority. In symptomatic upper-limb rehabilitation, the clinically relevant questions therefore remain whether BFRT provides additional benefit beyond appropriate active rehabilitation and how cuff pressure, exercise load, frequency, duration, progression, and co-interventions influence pain, strength, and functional outcomes.

Several reviews have examined upper-extremity BFRT and suggest potential clinical benefits, although the evidence remains limited and heterogeneous. Ponce-Fuentes et al reported improvements in selected patient-reported and physical-function outcomes but emphasized the limited quality of the available evidence.^[11]^ Johnson et al similarly suggested potential benefits for pain, strength, and patient-reported outcomes while highlighting the small evidence base.^[12]^ Jing et al found that low-load BFRT improved upper-extremity strength and hypertrophy, although their review included broader clinical and nonclinical populations.^[13]^ Sinnott et al also identified potential improvements in upper-extremity strength and hypertrophy but noted substantial variation in BFRT protocols.^[14]^ Chen et al reported reductions in pain and improvements in function and grip strength with BFRT in upper-extremity musculoskeletal disorders.^[15]^

The present review extends this literature through the prespecified cutoff of February 18, 2026 by incorporating more recent primary studies involving symptomatic and postoperative upper-limb musculoskeletal conditions^[19–23]^ and by emphasizing study-level effect estimates, both significant and nonsignificant comparative findings, BFRT prescription, co-interventions, and safety and tolerability.

The objective of this review was to evaluate changes in pain, strength, muscle morphology, and function; compare the effects of BFRT with active or sham comparators; and examine tolerability and prescription characteristics. By clarifying the clinical effects, prescription characteristics, and tolerability of BFRT, this review may help inform evidence-based integration of low-load BFRT into upper-limb rehabilitation practice and guide the design of future rehabilitation protocols.

## 2. Materials and methods

### 2.1. Protocol and registration

This systematic review was registered in the International Prospective Register of Systematic Reviews (PROSPERO; CRD420261299726), and its reporting was guided by the Preferred Reporting Items for Systematic Reviews and Meta-Analyses 2020 statement.^[24]^ The registered review start date was July 10, 2025, and the PROSPERO record was published on February 11, 2026. A full internal protocol was developed but was not publicly available.

### 2.2. Eligibility criteria

Eligibility was defined using a PICOS-based framework. Adults aged 18 years or older with upper-limb musculoskeletal conditions within the registered clinical domain, including LET/lateral epicondylitis, supraspinatus or rotator-cuff tendinopathy, subacromial impingement syndrome, and postoperative distal-radius-fracture rehabilitation, were eligible. Other adult symptomatic or postoperative upper-limb musculoskeletal conditions were considered when otherwise consistent with the registered clinical domain and when a defined musculoskeletal diagnosis and rehabilitation indication were reported. Eligible BFRT interventions included low-load resistance exercise, eccentric exercise, functional rehabilitation, or BFRT used as an identifiable adjunct to a broader rehabilitation program. No minimum or maximum BFRT treatment duration was prespecified. Co-interventions such as conventional resistance exercise, mobility or stretching, manual therapy, education, ultrasound, and electrical stimulation were permitted when the BFRT component could be identified.

Comparators could include sham BFR, conventional rehabilitation, high-load or low-load resistance exercise, no intervention, or pre–post comparison in uncontrolled case designs. For this review, conventional rehabilitation referred to the usual or standard rehabilitation program provided without BFRT, as described in the respective primary study, whereas resistance exercise referred specifically to strengthening exercise performed against resistance.

Eligible study designs were randomized controlled trials, case series, and case reports. At least 1 outcome related to pain, grip or other strength, muscle hypertrophy/morphology, function/disability, range of motion (ROM), neuromuscular performance, safety, or tolerability was required. Exclusion criteria were non-adult populations, conditions outside the registered upper-limb musculoskeletal scope, interventions without BFRT, animal or laboratory studies, non-original publications, non-English publications, ineligible outcomes, and reports unavailable in full text.

### 2.3. Information sources and search strategy

The registered strategy specified CENTRAL/Cochrane Library, Embase via Ovid, MEDLINE/PubMed, Science Citation Index/Web of Science, and Scopus. The database searches were performed by 2 authors (RS and AKA). No restriction was placed on the search start date, and eligible publications were required to be published or available as a citable online-1st article before February 18,2026; unpublished studies were not planned for inclusion. Backward reference-list checking was prespecified as an additional identification method. Explicit intervention terms included “blood flow restriction,” “blood flow restriction training,” “blood flow restriction exercise,” BFR, BFRT, KAATSU, “occlusion training,” and “vascular occlusion.” Upper-limb and clinical concepts included “upper extremity,” “upper limb,” shoulder, rotator cuff, supraspinatus, subacromial, elbow, epicondylitis, “tennis elbow,” “lateral elbow tendinopathy,” wrist, hand, biceps, “distal radius,” and postoperative upper-limb rehabilitation. Database-appropriate Boolean operators and subject headings were used. Platform-specific line-by-line historical search logs were not retained for every database and were therefore not reconstructed retrospectively during revision.

### 2.4. Study selection

Records from the historical electronic search were managed in Covidence. Two reviewers independently screened records in 2 stages: title/abstract screening followed by full-text eligibility assessment against the prespecified criteria. During peer-review revision, the retained study-selection record and available primary reports were reaudited against the registered criteria, with 1 primary protocol-based reason assigned to each full-text exclusion. The prespecified backward reference-list/citation process was also completed and cross-checked; reports identified through that pathway were assessed against the same eligibility criteria. All reports sought for the revision-stage eligibility assessment were successfully retrieved. Because the total number of references inspected during backward/reference-list checking could not be reconstructed from the retained historical audit trail, that denominator could not be reported reliably.

### 2.5. Data extraction

Two reviewers (RS and AKA) independently extracted data using a standardized extraction framework that captured study design, enrollment/randomization, analyzed or treated sample, participant condition, age and sex as reported, BFRT pressure/dosing approach, exercise load, repetition scheme, frequency, intervention duration, comparator/co-interventions, follow-up, quantitative outcomes, *P* values or confidence intervals where available, attrition, and adverse-event reporting. Extracted study-level data were cross-checked against the primary reports before synthesis. Across the 20 included reports, 340 participants were enrolled or randomized, and 326 contributed to the analyzed/treated clinical samples summarized in this review. Sex was reported for those 326 participants (158 men [48.5%] and 168 women [51.5%]); sex was not separately available for the 14 enrolled/randomized participants who did not contribute to those analyzed/treated samples. Study-level enrollment, analysis, and attrition are shown in Tables 1 and 2. Because age was reported as individual ages, group means ± SD, ranges, or median/interquartile range values, an entire-sample pooled mean ± SD age was not calculated without unsupported imputation.

**Table 1:** Characteristics and BFRT prescription of included studies.

Study/design/sample size	Clinical population/participant characteristics	BFRT protocol/comparator and co-interventions	Intervention duration/follow-up/outcomes
Wentzell, 2018^[20]^ Case report; n = 1	Postoperative distal biceps tendon reattachment; adult man; age reported as 30 yr in abstract and 35 yr in case text	BFR initiated postoperative week 3; fixed cuff pressure 80 mm Hg; 30-15-15-15; initially no external load, progressed gradually to 4 lb; comparator/co-interventions: multimodal postoperative rehabilitation; no control	15 wk; discharged at ~3.5 mo; outcomes: NPRS, DASH, Mayo Elbow Performance Index, ROM, function/return
Cancio et al. (2019)^[21]^ RCT; n = 13	Nonoperative DRF; mean age 46 yr (27–69); 6 M/7 F	Upper-arm tourniquet; individualized 50% occlusion; 30-15-15-15 during strengthening; comparator/co-interventions: standard hand-therapy rehabilitation without BFR	8 wk therapy; assessments to 14 wk post immobilization; outcomes: activity/rest pain, PRWE, DASH, ROM, grip/pinch, radiographs
Sgromolo et al. (2020)^[22]^ RCT; n = 9	Postoperative DRF; mean age 40 yr (22–54); 5 M/4 F	~50% LOP; 30-15-15-15; 2–3 sessions/wk; comparator/co-interventions: standard rehabilitation without BFR	8 wk; outcomes: pain, PRWE, ROM, grip/pinch, radiographs
Safford et al. (2021)^[23]^ Case report; n = 1	Adhesive capsulitis + rotator-cuff tendinopathy; 40-yr man	BFR adjunct; low-load resistance progressed clinically; comparator/co-interventions: multimodal conventional care; no control	7 visits over 13 wk; outcomes: ROM, strength, function
Ceballos et al. (2022)^[24]^ Case series; n = 2	Subacromial impingement/high irritability; 51-yr woman, 46-yr man	~20% maximal isometric strength; 50% LOP; 30-15-15-15; comparator/co-interventions: mobility and rehabilitation co-interventions; no control	3–4 visits over 3–4 wk; outcomes: pain, PPT, AROM, strength, PENN, ASES, PSFS
Karanasios et al. (2022)^[25]^ Sham-controlled RCT; n = 46	LET; 24 M/22 F; age reported by group	Low-load wrist-extensor training; ~40%–50% LOP; 30-15-15-15; 2 sessions/wk; comparator/co-interventions: same LLRT + sham BFR; massage, advice, home exercise	6 wk; follow-up 12 wk; outcomes: pain, PFGS, PRTEE, GROC
Magni et al. (2022)^[26]^ 3-arm feasibility RCT; n = 59	Symptomatic hand OA; BFR n = 19 age 67.9 ± 8.6; HIT n = 20 age 72.7 ± 9.0; advice n = 20 age 75.6 ± 12.0; 49 F/10 M overall	30%–40% MVC/1RM; 50% LOP; 30-15-15-15 progression; 3 sessions/wk; comparator/co-interventions: high-intensity training + advice; advice only	6 wk; outcomes: pain/flare, grip, hand function, treatment response, feasibility
Miller et al. (2022)^[27]^ Two case reports; n = 2	Subacromial pain; women aged 27 and 28 yr	~20% maximal isometric strength; 50% occlusion; 30-15-15-15; comparator/co-interventions: conventional rehabilitation; no control	Case A: 16 visits over 8 wk; case B: 22 visits over 11 wk; outcomes: pain, ROM, strength, muscle CSA, function/performance
McGinniss et al. (2022)^[28]^ Prospective case series; 23 enrolled; 20 completed BFR/12-wk analysis; 18 completed 6-mo follow-up	Postoperative shoulder stabilization; principal 6–12-wk analytic sample age 21.3 ± 1.9 yr; 17 M/3 F	BFR from post-op week 6; ~50% LOP; 30-15-15-15; ~2–3/wk; comparator/co-interventions: standard postoperative rehabilitation; no control	6 wk BFR; follow-up to 6 mo; outcomes: shoulder strength, SANE/SPADI, ROM, performance
Lorenz (2022)^[29]^ Case report; n = 1	Postoperative distal biceps repair; 39-yr man	Progressive loading with BFR/ischemic preconditioning; comparator/co-interventions: individualized postoperative loading; no control	Follow-up ~6 mo; outcomes: isokinetic strength, advanced loading, return
Fan et al. (2023)^[30]^ RCT; 40 randomized/35 analyzed	Postoperative DRF; analyzed sample mean age 46 yr (25–74); 17 M/18 F	Fixed 120 mm Hg; ~20 min; 5 sessions/wk; 30-15-15-15; comparator/co-interventions: same 4-wk rehabilitation without BFR	4 wk; selected follow-up 12 wk; outcomes: VAS, wrist strength, ROM, swelling, D-dimer, RUSS, Cooney
Poursaleh Begi et al. (2023)^[31]^ Case report; n = 1	Supraspinatus tendinopathy; 40-yr man	12 sessions (~3 sessions/wk); 30-15-15-15; load progressed ~15%–20% to 30%–40% 1RM; pressure progressed from ~30%–40% to 50% LOP; comparator/co-interventions: rotator-cuff/scapular program; no control	~4 wk; outcomes: pain, PPT, muscle thickness, strength
Karanasios et al. (2023)^[32]^ Case report; n = 1	Chronic LET; 51-yr man	Wrist-extensor BFR ~40% AOP/LOP; 30-15-15-15; 12 visits; comparator/co-interventions: multimodal rehabilitation; no control	6 wk; follow-up 12 wk; outcomes: NPRS, PFGS, PRTEE, GROC, PPT
Kinandana et al. (2023)^[33]^ Randomized study; 28 randomized/24 analyzed	Lateral epicondylitis; analyzed-group ages 46.00 ± 3.08 and 45.83 ± 2.73; 15 M/9 F among 24 analyzed; sex unavailable for 4 randomized	20%–40% 1RM; pressure ~1.2 × systolic BP; 4 × 12; 3 sessions/wk; comparator/co-interventions: high-intensity resistance 70%–80% 1RM; both groups ultrasound + electrical stimulation	4 wk; outcomes: grip strength; patient-rated tennis elbow evaluation questionnaire (PrTEEQ)
Kara et al. (2024)^[34]^ Assessor-blinded RCT; n = 28	Rotator-cuff tendinopathy; 14 M/14 F; group mean ages 31.1 and 34.9 yr	Low-load BFR at 50% LOP; 2 sessions/wk; comparator/co-interventions: Identical rehabilitation without BFR	8 wk; outcomes: muscle thickness, isokinetic strength, VAS, SPADI
Ponce-Fuentes et al. (2024)^[35]^ Case report; n = 1	After arthroscopic rotator-cuff repair; 54-yr man	Early postoperative low-intensity BFRT; comparator/co-interventions: postoperative rehabilitation; no control	12 wk; follow-up 6 mo; outcomes: pain, ROM, strength, muscle measures, function
Ponce-Fuentes et al. (2025)^[36]^ Case report; n = 1	Postoperative olecranon fracture; 27-yr man	50% LOP; 75 repetitions/exercise; low-load resistance; comparator/co-interventions: postoperative rehabilitation; no control	12 wk; outcomes: pain, elbow/forearm ROM, grip, function/disability
Karanasios et al. (2025) online^[37]^ Randomized crossover; n = 20	LET; age 49.6 ± 6.6 yr; 12 M/8 F	Single wrist-extensor session; 30-15-15-15 with BFR; comparator/co-interventions: same exercise without BFR; conditions 1 wk apart	Single session/condition; outcomes: PPT, PFGS, BP, perceived exertion
Lear et al. (2025) online^[38]^ Open-label RCT; n = 41	Lateral epicondylitis; median age 52 yr (IQR 42–58); 19 M/22 F	Low-load BFR physical therapy; once-weekly supervised program; comparator/co-interventions: standard heavy-load physical therapy + home exercise	6 wkly visits; follow-up 12 mo; outcomes: PRTEE, NPRS, pain-free and maximum grip
Park et al. (2025/2026) online issue^[39]^ Pilot single-blind RCT; 22 randomized/20 analyzed	Chronic lateral epicondylitis; analyzed sample 11 M/9 F; both groups mean age 34 yr	Upper-arm pneumatic cuff; individualized 40%–50% AOP; same progressive low-load exercise program as control; comparator/co-interventions: conventional physical therapy + identical exercise program without cuff	6 wk; 2 supervised sessions/wk; outcomes: NPRS, PPT, ECRB pennation angle, PFGS, WES, PRTEE, QuickDASH, FABQ, SF-12, adverse events

AOP = arterial occlusion pressure, AROM = active range of motion, ASES = American Shoulder and Elbow Surgeons, BFR = blood flow restriction, BP = blood pressure, CSA = cross-sectional area, DASH = disabilities of the arm, shoulder and hand, DRF = distal radius fracture, ECRB = extensor carpi radialis brevis, F = female, FABQ = fear-avoidance beliefs questionnaire, GROC = global rating of change, HIT = high-intensity training, LET = lateral elbow tendinopathy, LOP = limb occlusion pressure, M = male, MVC = maximal voluntary contraction, OA = osteoarthritis, PENN = penn shoulder score, PFGS = pain-free grip strength, PPT = pressure pain threshold, PRTEE = patient-rated tennis elbow evaluation, PrTEEQ = patient-rated tennis elbow evaluation questionnaire, PRWE = patient-rated wrist evaluation, PSFS = patient-specific functional scale, QuickDASH = quick disabilities of the arm, shoulder and hand, RCT = randomized controlled trial, ROM = range of motion, SF-12 = 12-item short form health survey, SPADI = shoulder pain and disability index, VAS = visual analog scale, WES = wrist-extensor strength.

**Table 2:** Quantitative outcomes, comparative findings, and safety/attrition of studies included in this review.

Study	Quantitative and comparative findings	Safety/attrition	Main outcomes
Wentzell (2018)^[20]^	NPRS 5/10–0/10; DASH primary 87.5–5; DASH work 100–6.25; DASH sport 100–25; Mayo Elbow Performance Index 20–95. Single-patient multimodal postoperative rehabilitation; no comparator group.	Adverse-event reporting was not explicitly standardized in the case report.	↓ Pain and disability; ↑ elbow function
Cancio et al. (2019)^[21]^	Mean activity-pain reduction 4.0 vs 2.3 points; mean PRWE reduction 57.9 vs 30.8 points. Greater activity-pain and PRWE reductions were reported with BFRT over 8 wk; radiographic outcomes were similar.	n = 13; therapy tolerated; no complications reported.	↓ Activity pain; ↓ PRWE
Sgromolo et al. (2020)^[22]^	Activity-pain interval change 3.0 vs 1.5; PRWE interval change 36.4 vs 18.8. Greater interval changes in activity pain and PRWE were reported with BFRT; DASH, grip/pinch, and ROM were not significantly different.	n = 9; all completed treatment; no complications or radiographic instability reported.	↓ Activity pain; ↓ PRWE; no significant differences in DASH, grip/pinch, or ROM
Safford et al. (2021)^[23]^	ROM and function normalized and shoulder strength increased approximately 60%–85% during a single-patient multimodal rehabilitation course; no comparator group.	No BFRT adverse effect reported.	↑ ROM, strength, and function
Ceballos et al. (2022)^[24]^	Patient-reported outcomes, PPT, ROM, and strength changed during treatment; 1 patient gained 30° flexion and 60° abduction. Two-patient case series; no comparator group.	Safety reporting not standardized.	Improvements in patient-reported outcomes, PPT, ROM, and strength
Karanasios et al. (2022)^[25]^	Pain at 12 wk: between-group difference −1.54 (95% CI −2.89 to −0.18); PFGS ratio at 6 weeks: +0.20 (0.06–0.34); PRTEE: −11.92 at 6 wk and −15.23 at 12 wk. These between-group estimates favored BFRT vs sham-BFR at the reported time points.	No major safety signal reported.	↓ Pain; ↑ PFGS; ↓ PRTEE
Magni et al. (2022)^[26]^	Responders: BFRT 15/19 (79%) vs advice 7/20 (35%); OR 6.96 (95% CI 1.66–29.3). Hand-pain change was −2.3 with BFRT vs +0.3 with advice; clinically important grip-strength and hand-function gains were not demonstrated.	BFRT retention 89%; pain flares in 1.6% of BFRT sessions; no BFRT adverse event; 1 HIT withdrawal because of pain.	↑ Treatment response; ↓ hand pain; no clinically important improvement in grip strength or hand function
Miller et al. (2022)^[27]^	Both cases showed pre–post changes in pain/function, ROM, strength, muscle CSA, and performance during multimodal rehabilitation; no comparator group.	No adverse events reported.	Reduced pain; improvements in function, ROM, strength, muscle CSA, and performance
McGinniss et al. (2022)^[28]^	From 6 to 12 wk: external rotation + 0.049 (95% CI 0.021–0.077), abduction + 0.079 (0.050–0.108), internal rotation + 0.060 (0.028–0.093), SANE + 17.7 (9.4–25.9), SPADI −31.1 (−44.2 to −18.0). Uncontrolled postoperative case series with concomitant rehabilitation.	23 enrolled; 20 completed the BFR program/12-wk analysis; 18 completed 6-mo follow-up. No adverse events or BFR-related discontinuations; 5 required initial pressure reduction from 50% to 40% LOP because of moderate discomfort.	↑ Shoulder strength and SANE; ↓ SPADI
Lorenz (2022)^[29]^	Symmetrical isokinetic elbow-flexor strength was described at approximately 6 mo during progressive postoperative loading; single case, no comparator group.	Adverse-event reporting was not explicit in the case report.	Restoration of symmetrical elbow-flexor strength
Fan et al. (2023)^[30]^	VAS week 2: 0.65 ± 0.79 vs 2.50 ± 0.62; week 4: 0.06 ± 0.24 vs 1.28 ± 0.67; Cooney score at 12 wk: 87.35 ± 4.00 vs 73.61 ± 5.64. Wrist flexion/extension strength also differed between groups; passive ROM did not.	40 randomized; 35 analyzed. No significant D-dimer or RUSS disadvantage reported.	↓ Pain; ↑ wrist flexion/extension strength; ↑ Cooney function; no significant difference in passive ROM
Poursaleh Begi et al. (2023)^[31]^	Pain 3.58–2.08; supraspinatus strength 3.16–7.23 N; thickness 8.41–8.64 mm; lower-trapezius strength 5.80–4.90 N. Single-patient pre–post findings; no comparator group.	Standardized adverse-event monitoring not established.	↓ Pain; ↑ supraspinatus strength; minimal ↑ muscle thickness; ↓ lower-trapezius strength
Karanasios et al. (2023)^[32]^	NPRS 7/10–0/10; PRTEE 48–0; PFGS ratio 0.73–1.03; PPT 7.5–9.5 kg/cm^2^ during multimodal rehabilitation. Single-patient report; no comparator group.	No adverse event reported.	↓ Pain and PRTEE; ↑ PFGS and PPT
Kinandana et al. (2023)^[33]^	Grip-strength change 10.93 vs 4.82; disability change also differed between groups. Both groups received ultrasound and electrical stimulation; 4 of 28 randomized participants were not analyzed.	28 randomized; 24 analyzed.	↑ Grip strength; ↓ disability
Kara et al. (2024)^[34]^	Biceps-brachii thickness and internal-rotation strength at 60°/s showed between-group differences favoring BFRT; pain and SPADI did not show significant between-group superiority.	No serious BFRT-related event reported.	↑ Biceps-brachii thickness and internal-rotation strength; no significant difference in pain or SPADI
Ponce-Fuentes et al. (2024)^[35]^	Pre–post changes in pain, ROM, strength/muscle measures, and function were reported during postoperative rehabilitation. Single case with concomitant rehabilitation; no comparator group.	Adverse-event reporting was not sufficiently detailed for a definite appraisal judgment.	Reduced pain; improvements in ROM, strength/muscle measures, and function.
Ponce-Fuentes et al. (2025)^[36]^	Pain 5.9–1.4 cm; elbow flexion 88°–137°; grip 8–47 kg; OES total 22.9–89.6 during postoperative rehabilitation. Single case; no comparator group.	Minor discomfort during BFRT; no adverse events were reported.	↓ Pain; ↑ ROM, grip strength, and function
Karanasios et al. (2025) online^[37]^	No significant PPT difference between conditions. The overall affected-side PFGS intervention × time interaction was *P* = .06; the adjusted post-intervention PFGS difference was 4.4 (95% CI 1.9–6.9) favoring BFR.	No significant BP or perceived-exertion difference.	No significant difference in PPT; ↑ post-intervention PFGS with BFR
Lear et al. (2025) online^[38]^	12-mo PRTEE change: BFRPT −30.54 vs standard PT −26.22; reported adjusted between-group contrast −4.32 (95% CI −9.17 to 17.81). NPRS and grip-strength between-group comparisons were also nonsignificant.	41 included in intention-to-treat analysis.	No significant differences in PRTEE, pain, or grip strength
Park et al. (2025/2026) online issue^[39]^	PRTEE change −24.20 ± 4.02 vs −15.60 ± 3.71; QuickDASH change −12.83 ± 4.97 vs −7.51 ± 4.72; FABQ change −5.30 ± 3.52 vs −2.50 ± 1.84. Between-group changes in NPRS, PPT, PFGS, wrist-extensor strength, pennation angle, and SF-12 mental component were not significant.	22 randomized; 20 analyzed per protocol. One scheduling withdrawal in BFRT; 1 worsening-pain withdrawal in control; no intervention-related adverse events reported.	↓ PRTEE, QuickDASH, and FABQ; improved SF-12 physical component; no significant differences in pain or strength

BFR/BFRT = blood flow restriction/training, BFRPT = blood flow restriction physical therapy, BP = blood pressure, CI = confidence interval, CSA = cross-sectional area, ER = external rotation, FABQ = fear-avoidance beliefs questionnaire, HIT = high-intensity training, IQR = interquartile range, IR = internal rotation, NPRS = numeric pain rating scale, OES = Oxford elbow score, OR = odds ratio, PFGS = pain-free grip strength, PPT = pressure pain threshold, PRTEE = patient-rated tennis elbow evaluation, PRWE = patient-rated wrist evaluation, QuickDASH = quick disabilities of the arm, shoulder and hand, RCT = randomized controlled trial, RUSS = radius union scoring system, SANE = single assessment numeric evaluation, SF-12 = 12-item short form health survey, SPADI = shoulder pain and disability index, SPT = standard physical therapy, VAS = visual analog scale, WES = wrist-extensor strength.

Extraction and synthesis explicitly distinguished controlled between-group effects from uncontrolled within-patient change. When several follow-up time points were reported, the narrative synthesis prioritized the main clinically relevant follow-up reported in the primary study, while additional time points were retained when needed to characterize the magnitude, direction, or statistical significance of the findings accurately.

### 2.6. Quality assessment

Two reviewers independently performed the design-specific risk-of-bias or critical appraisal for each included report. Parallel-group randomized trials were evaluated using the Cochrane risk of bias 2 (RoB 2) tool, and the randomized crossover trial was evaluated using the RoB 2 crossover-trial variant.^[25]^ RoB 2 judgments were anchored to the principal clinical result at the main reported follow-up and used the standard categories low risk, some concerns, and high risk. Case series were appraised with the Joanna Briggs Institute (JBI) Critical Appraisal Checklist for Case Series,^[26]^ and case reports with the JBI Critical Appraisal Checklist for Case Reports.^[27]^ The registered PROSPERO record had specified the CAse REport (CARE) guideline for case reports; during revision, the JBI case-report checklist was used instead for methodological critical appraisal because CARE is a reporting guideline rather than a risk-of-bias tool. This revision-stage change is therefore reported transparently as a protocol deviation. JBI item-level responses were recorded as Yes, No, Unclear, or Not applicable. When reporting did not support a definite appraisal judgment, uncertainty was recorded rather than inferred. No numerical quality score or nonstandard High/Moderate/Low JBI summary category was generated. Methodological limitations were incorporated into the narrative interpretation.

### 2.7. Data synthesis

A meta-analysis was not performed because diagnoses, study designs, comparators, BFRT prescriptions, outcome instruments, and follow-up intervals were clinically and methodologically heterogeneous. Findings were therefore synthesized narratively by outcome domain, with emphasis on magnitude, between-group effects, clinical relevance where the primary study permitted such interpretation, intervention prescription, and safety/tolerability. No subgroup analysis, sensitivity analysis, formal assessment of reporting bias, or certainty-of-evidence grading was undertaken because no pooled effect estimate or common quantitative synthesis framework was considered defensible across the mixed designs and heterogeneous outcomes.

### 2.8. Ethics and consent

This systematic review analyzed previously published studies and involved no new participant recruitment, collection of identifiable human data, or intervention in patient care. Institutional review board or ethics committee approval and new informed consent were therefore not required for this review.

## 3. Results

### 3.1. Study selection

The electronic database search identified 183 records. After removal of 149 duplicates, 34 records underwent title/abstract screening, and 9 were excluded. Twenty-five reports were retrieved and assessed for eligibility; 20 were excluded because of an ineligible population/condition (n = 6), intervention (n = 4), study design/publication type (n = 5), or outcome (n = 5), leaving 5 eligible reports from the database search. Backward reference-list/citation checking identified 16 additional potentially eligible reports; 1 was excluded because the population was outside the prespecified clinical scope (healthy adults without an upper-limb musculoskeletal condition), and 15 were included. Overall, 20 reports met the eligibility criteria and were included in the systematic review (Fig. 1). Park et al was eligible because a citable online-1st version was available on December 22, 2025, before the prespecified cutoff of February 18, 2026.

**Figure 1.**
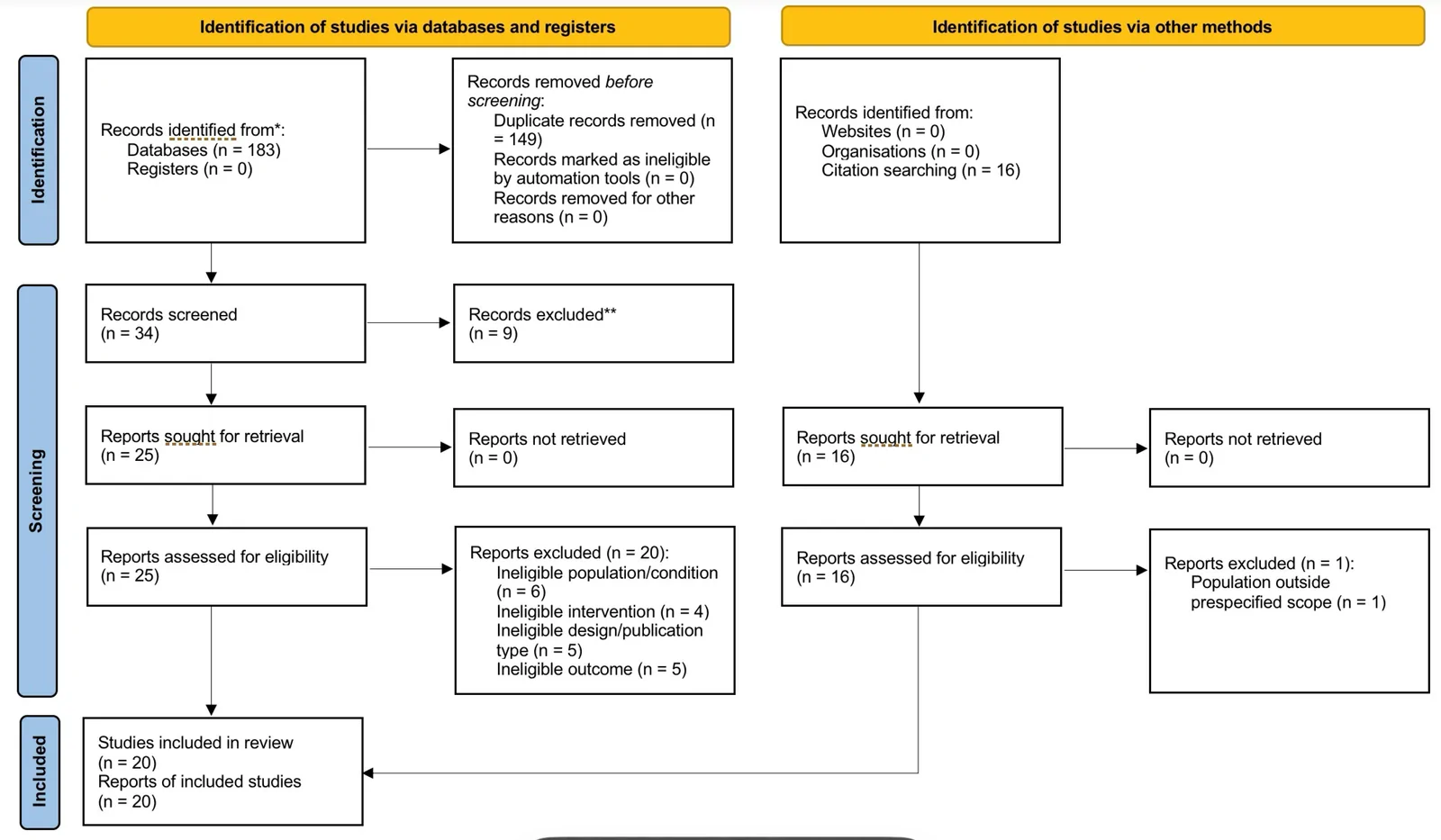
PRISMA 2020 flow diagram. PRISMA = Preferred Reporting Items for Systematic Reviews and Meta-Analyses.

### 3.2. Characteristics of included studies

Twenty reports were included: 9 parallel-group randomized trials, 1 randomized crossover trial, 2 case series, and 8 case-report publications.^[16–23,28–39]^ Ten of the 20 included reports (50%) were case reports or case series, underscoring the limited size and maturity of the current evidence base. Across all reports, 340 participants were enrolled or randomized, and 326 contributed to the analyzed or treated samples. Among these 326 participants, 158 were men and 168 were women. Clinical populations included distal radius fracture (DRF), lateral elbow tendinopathy/lateral epicondylitis, rotator cuff or supraspinatus disorders, subacromial pain/impingement, hand osteoarthritis, postoperative shoulder stabilization, distal biceps repair, postoperative rotator cuff repair, and postoperative olecranon fracture. Participant age was reported heterogeneously and is presented at the study level. Table 1 summarizes the study characteristics and BFRT prescriptions.

### 3.3. Results of included studies

#### 3.3.1. Lateral elbow tendinopathy/epicondylitis

Among studies of lateral elbow tendinopathy, Karanasios et al randomized 46 patients to 6 weeks of supervised low-load resistance training at 30% 1RM with BFR at 40% to 50% arterial occlusion pressure (AOP), performed twice weekly, or the same program with sham BFR.

BFRT resulted in a significantly greater reduction in pain at 12 weeks (between-group difference −1.54, 95% confidence interval [CI] −2.89 to −0.18; *P* = .026), a greater increase in pain-free grip strength (PFGS) ratio at 6 weeks (+0.20, 95% CI 0.06 to 0.34; *P* = .005), and greater reductions in patient-rated tennis elbow evaluation (PRTEE) at 6 and 12 weeks (−11.92, 95% CI −20.26 to −3.59; *P* = .006; and −15.23, 95% CI −23.57 to −6.90; *P* < .001, respectively).^[16]^ The 12-week PRTEE difference exceeded the >11-point threshold described by the trial authors as clinically significant. Park et al randomized 22 patients with chronic lateral epicondylitis, of whom 20 completed the study, to 6 weeks of twice-weekly conventional physical therapy with or without BFRT at 40% to 50% of individual AOP. Reductions in PRTEE (−24.20 ± 4.02 vs −15.60 ± 3.71; *P* = .001) and quick disabilities of the arm, shoulder and hand (−12.83 ± 4.97 vs −7.51 ± 4.72; *P* = .025) were significantly greater with BFRT, whereas changes in numeric pain rating scale, PFGS, and wrist-extensor strength did not differ significantly between groups.^[19]^ In 41 patients with lateral epicondylitis, Lear et al compared up to 6 weekly physical therapy visits using low-load wrist-extension exercise at 20% 1RM with BFR at 50% LOP with standard physical therapy using wrist-extension exercise at 70% 1RM. Both groups showed reductions in PRTEE at 12 months, but the between-group difference was not significant (adjusted contrast + 4.32, 95% CI −9.17 to 17.81; *P* = .53); numeric pain rating scale and grip-strength differences were also not significant.^[23]^ In a randomized crossover trial of 20 patients, a single session of wrist-extensor exercise using a 30-15-15-15 repetition scheme with BFR at 40% to 50% LOP was compared with the same exercise without BFR. Pressure-pain-threshold changes did not differ significantly between conditions (*P* > .05), and the overall intervention-by-time interaction for affected-side PFGS was not significant (*P* = .06), although adjusted post-intervention PFGS was significantly greater with BFR by 4.4 (*P* = .001).^[22]^

Kinandana et al randomized 28 patients with lateral epicondylitis to 4 weeks of low-load BFRT at 20% to 40% 1RM or high-intensity resistance exercise at 70% to 80% 1RM, performed 3 times/wk; both groups also received ultrasound and electrical stimulation. Among 24 analyzed participants, the increase in grip strength and reduction in disability were significantly greater with BFRT (*P* < .001).^[39]^

#### 3.3.2. Distal radius fracture rehabilitation

Three randomized studies assessed the efficacy of BFRT during DRF rehabilitation. In 13 patients with nonoperatively managed DRFs, Cancio et al compared 8 weeks of standardized rehabilitation with or without BFRT at 50% of maximal LOP, performed 2 to 3 times/wk. BFRT resulted in significantly greater reductions in activity pain (4.0 vs 2.3 points; *P* = .03) and patient-rated wrist evaluation (PRWE) score (57.9 vs 30.8 points; *P* = .01), while radiographic outcomes did not differ significantly between groups.^[29]^ Sgromolo et al randomized 9 patients following operative DRF management to BFRT plus standardized rehabilitation (n = 5) or the same standardized rehabilitation without BFR (n = 4). The BFRT group completed the 8-week protocol at 50% maximal LOP 2 to 3 times/wk. BFRT resulted in significantly greater reductions in activity pain (3.0 vs 1.5 points; *P* = .03) and PRWE (36.4 vs 18.8 points; *P* = .01), whereas ROM, disabilities of the arm, shoulder and hand, grip strength, and pinch strength did not differ significantly between groups.^[30]^ Fan et al randomized 40 postoperative patients, of whom 35 were analyzed, to 4 weeks of regular rehabilitation with or without BFRT performed 5 times/wk at 120 mm Hg, with low-load strengthening during the program. BFRT resulted in significantly lower visual analog scale pain at weeks 2 and 4 (*P* < .01), greater wrist flexion and extension strength (*P* < .001), and higher 12-week Cooney function scores (*P* < .01), whereas passive ROM did not differ significantly between groups.^[17]^

#### 3.3.3. Hand osteoarthritis

In 59 patients with symptomatic hand osteoarthritis, Magni et al compared 6 weeks of supervised BFRT plus advice, high-intensity resistance training plus advice, and advice alone; the exercise groups trained 3 times/wk, with BFRT performed at 30% to 40% maximal voluntary contraction and 50% LOP. At 6 weeks, 15/19 participants receiving BFRT (79%) met the Outcome Measures in Rheumatology–Osteoarthritis Research Society International treatment-response criteria compared with 7/20 participants (35%) receiving advice alone (odds ratio 6.96, 95% CI 1.66–29.3; *P* = .01). Pain decreased by 2.3 points with BFRT compared with a 0.3-point worsening with advice alone, whereas clinically important improvements in grip strength and function were not demonstrated.^[33]^

#### 3.3.4. Rotator cuff and shoulder conditions

In 28 patients with rotator-cuff tendinopathy, Kara et al compared 8 weeks of twice-weekly low-load exercise at 30% 1RM with or without BFR at 50% LOP. BFRT resulted in significantly greater biceps-brachii thickness (*P* = .002) and internal-rotation strength at 60°/s (*P* = .040), while pain and shoulder pain and disability index (SPADI) did not differ significantly between groups.^[18]^

#### 3.3.5. Other postoperative and case-based upper-limb conditions

Uncontrolled studies also reported improvements across several upper-limb conditions, although these changes cannot be attributed to BFRT alone. In the shoulder-stabilization case series, 23 postoperative patients were enrolled, and 20 completed BFRT beginning at postoperative week 6, performed 2 to 3 times/wk at 50% LOP in addition to standard rehabilitation. From weeks 6 to 12, normalized external-rotation strength increased by 0.049 kg/kg body mass, abduction strength by 0.079 kg/kg body mass, and internal-rotation strength by 0.060 kg/kg body mass; single assessment numeric evaluation increased by 17.7 points and SPADI decreased by 31.1 points (all *P* < .001).^[35]^ In 2 patients with highly irritable subacromial impingement, BFRT was added to low-load shoulder strengthening using a 30-15-15-15 repetition scheme, with reductions in pain and improvements in strength and patient-reported function reported during rehabilitation.^[32]^ Two additional patients with subacromial pain completed BFRT at 50% maximal occlusion pressure using resistance equivalent to 20% of maximal isometric strength; both demonstrated improvements in shoulder function, ROM, strength, and muscle morphology.^[34]^

Among individual case reports, a patient with lateral elbow tendinopathy completed 6 weeks (12 visits) of wrist-extensor BFRT at 40% AOP as part of multimodal rehabilitation; pain decreased from 7/10 to 0/10, PRTEE from 48 to 0, and PFGS ratio increased from 0.73 to 1.03.^[38]^ A patient with supraspinatus tendinopathy completed 12 BFRT sessions over 4 weeks using low-load exercise at 20% to 40% 1RM and 50% LOP; pain decreased from 3.58 to 2.08 and supraspinatus strength increased from 3.16 to 7.23 N, whereas lower-trapezius strength decreased from 5.80 to 4.90 N.^[37]^ In a patient with adhesive capsulitis and rotator-cuff tendinopathy, BFRT was incorporated into 7 rehabilitation visits over 13 weeks, with normalization of shoulder ROM and function and 60% to 85% strength gains.^[31]^ Wentzell introduced BFRT during the 3rd postoperative week after distal-biceps reattachment using a 30-15-15-15 repetition scheme at 80 mm Hg, with progressive improvements in ROM, strength, and function during rehabilitation.^[28]^ Lorenz used BFRT during progressive rehabilitation after distal-biceps repair, including a 30-15-15-15 repetition scheme when isolated biceps strengthening began at postoperative week 6; symmetrical isokinetic elbow-flexor strength was achieved by 6 months.^[36]^ In a patient following arthroscopic rotator-cuff repair, a 12-week postoperative program incorporating low-intensity BFRT was associated with clinically meaningful reductions in pain and improvements in muscle thickness, shoulder strength, ROM, and function.^[20]^ In a patient following operative olecranon fracture management, 12 weeks of rehabilitation included BFRT at 50% LOP with 75 repetitions per exercise; reductions in pain and disability and improvements in elbow and forearm ROM, grip strength, and function were reported.^[21]^

#### 3.3.6. Safety and tolerability

Safety and tolerability reporting was heterogeneous. No BFRT-related adverse events or complications were reported in several included studies.^[19,29,30,33,35]^ Fan et al found no significant between-group differences in D-dimer levels or radius union scoring system scores.^[17]^ Five participants in McGinniss et al required an initial reduction in pressure from 50% to 40% LOP because of moderate discomfort.^[35]^ Several case-based reports also explicitly reported no BFRT-related adverse events, whereas adverse-event ascertainment was not systematically described in several other reports. Table 2 provides the quantitative outcomes, comparative findings, safety, attrition, and statistical results.

### 3.4. Quality assessment of included studies

All 20 reports underwent design-specific appraisal. Among the 9 parallel-group randomized trials, 1 sham-controlled trial was judged low risk across RoB 2 domains, 7 raised some concerns overall, and 1 was judged high risk overall because analyzed-sample loss and reporting limitations materially reduced confidence in the principal clinical result^[16–19,23,29,30,33,39]^ (Fig. 2). The randomized crossover trial raised some concerns overall, mainly because sequence-specific period/carryover protections and prespecification details were incompletely reported^[22]^ (Fig. 3). The 2 case series were appraised with the 10-item JBI checklist; reporting was generally adequate for participant characteristics, interventions, and outcomes, while consecutive/complete inclusion and site-level reporting were unclear in 1 or both reports^[32,35]^ (Fig. 4). The 8 case-report publications were appraised with the 8-item JBI checklist. Intervention and post-intervention clinical course were generally described, while uncertainties included the internally inconsistent patient age and non-explicit adverse-event reporting in Wentzell, incomplete diagnostic-assessment detail in Safford, non-explicit adverse-event reporting in Lorenz, history/adverse-event reporting in Poursaleh Begi, and adverse-event detail in Ponce-Fuentes 2024^[20,21,28,31,34,36–38]^ (Fig. 5). Where reporting was insufficient, the item was rated conservatively rather than inferred. These item-level appraisals were not converted into numerical or nonstandard overall JBI quality scores.

**Figure 2.**
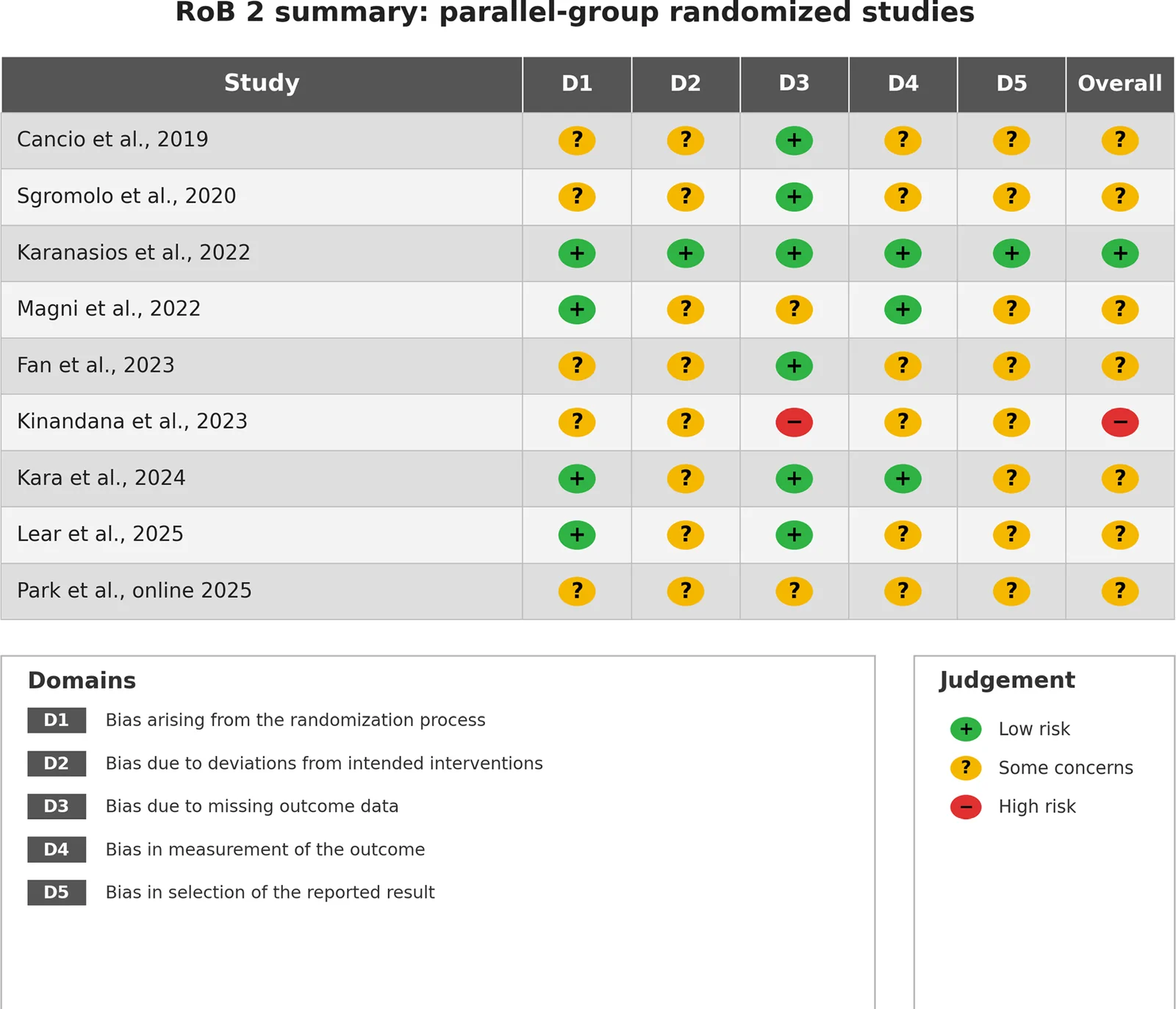
Cochrane RoB 2 assessment for parallel-group randomized studies, anchored to the principal clinical result at the main reported follow-up. RoB 2 = risk of bias 2.

**Figure 3.**
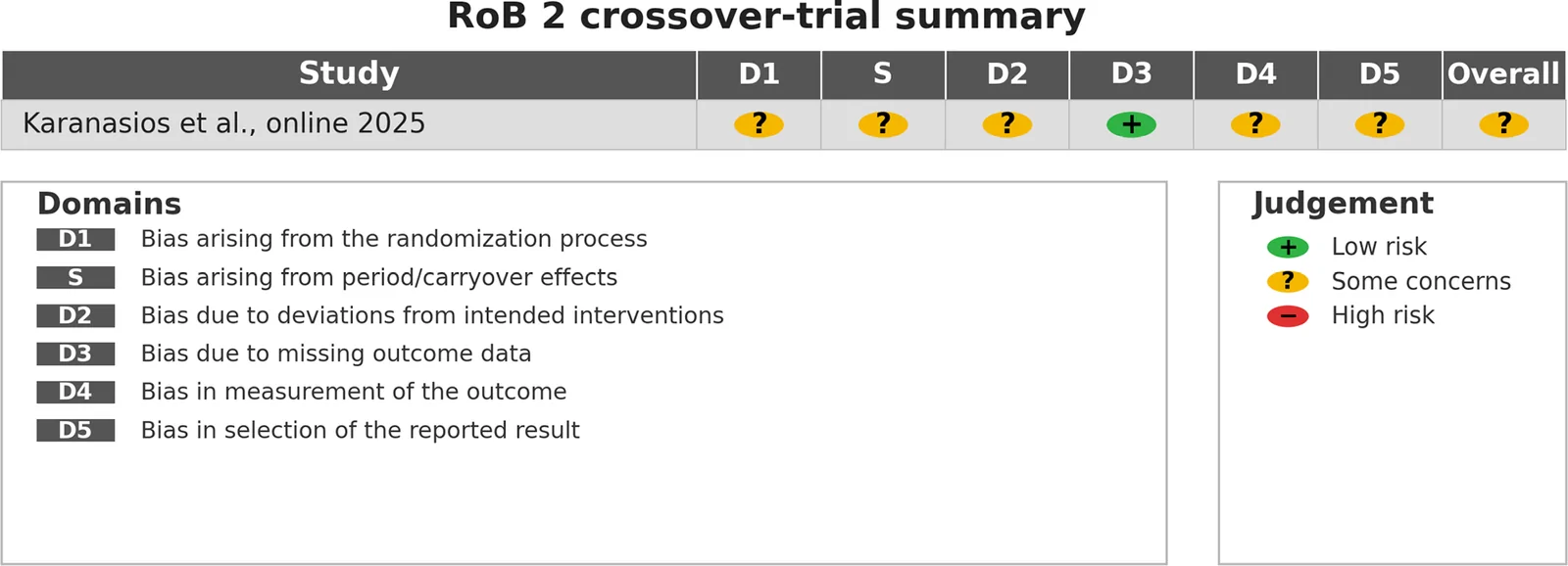
RoB 2 crossover-trial assessment for the randomized crossover study, including the period/carryover domain. RoB 2 = risk of bias 2.

**Figure 4.**
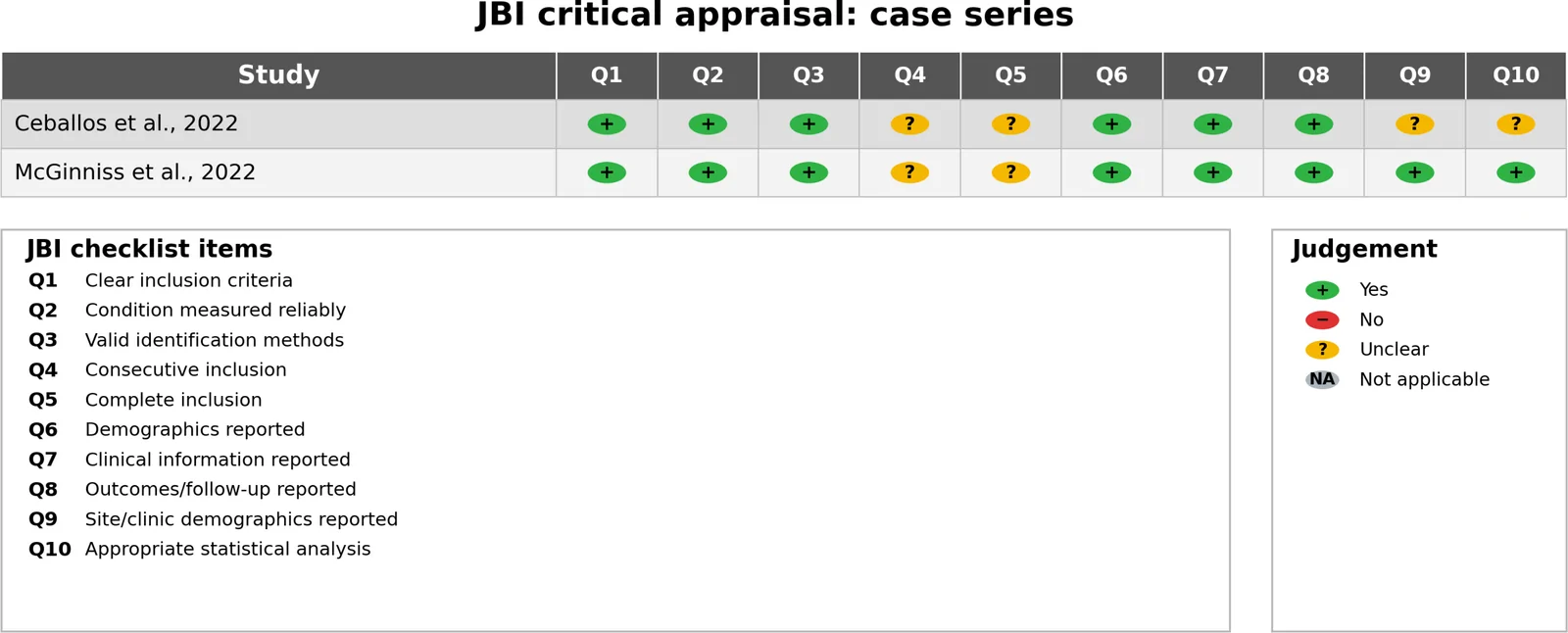
JBI critical appraisal of case series. Item-level responses only; no numerical or nonstandard overall quality score was assigned. JBI = Joanna Briggs Institute.

**Figure 5.**
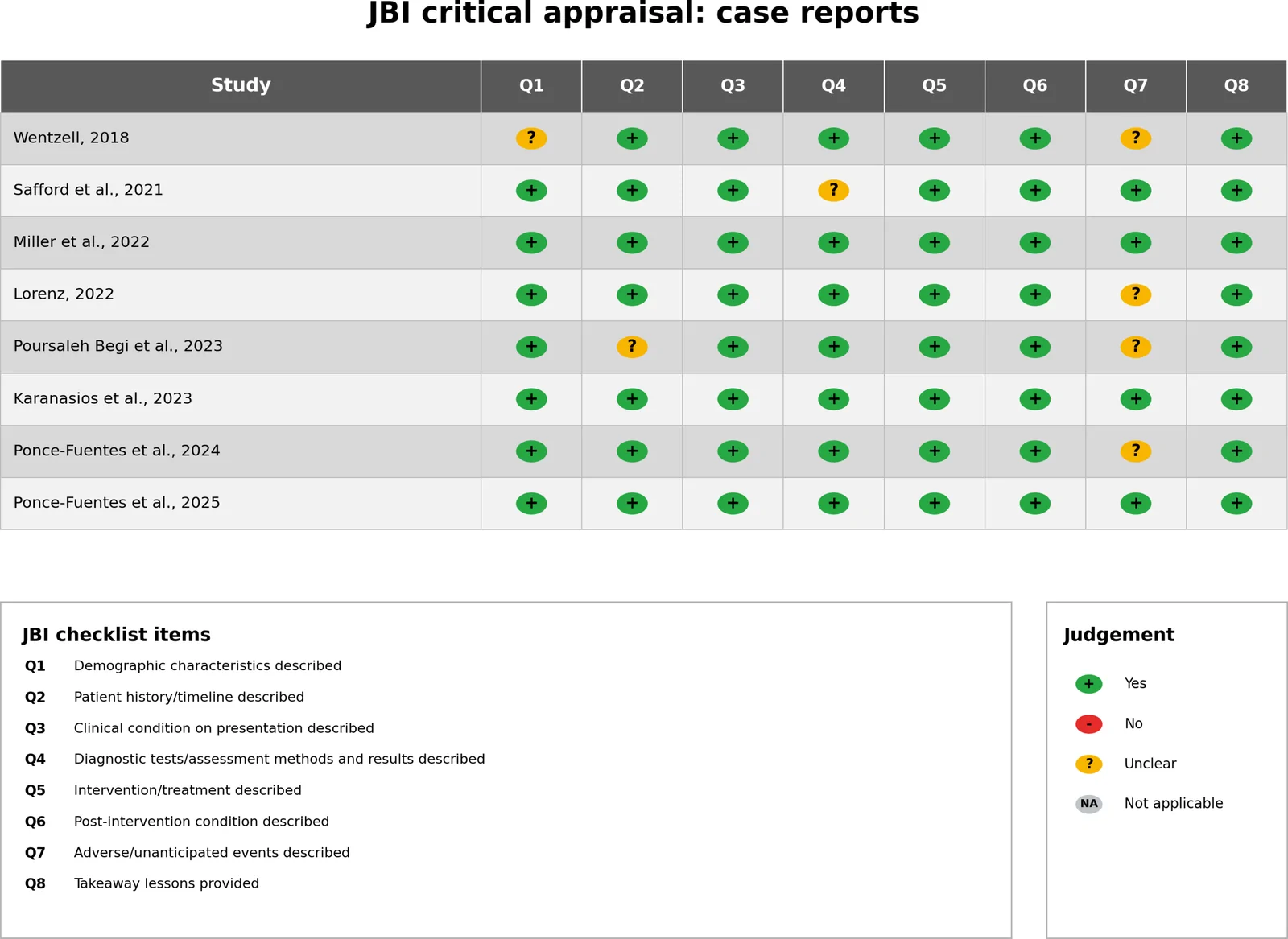
JBI critical appraisal of case-report publications. Item-level responses only; no numerical or nonstandard overall quality score was assigned. JBI = Joanna Briggs Institute.

## 4. Discussion

The aim of this systematic review was to evaluate the effects of BFRT on pain, strength, muscle morphology, and function in adult upper-limb musculoskeletal rehabilitation, while also examining tolerability and prescription characteristics. Overall, BFRT showed significant benefits for selected pain, disability, strength, muscle morphology, and early postoperative outcomes in some randomized trials, but these effects were not consistent across conditions, outcomes, or comparators. The evidence therefore suggests that BFRT may be a useful low-load adjunct in selected upper-limb conditions, but it is not consistently superior to conventional physical therapy or resistance-training interventions without BFR. The comparative evidence for lateral elbow tendinopathy suggests that the effects of BFRT depend on the outcome measured and the comparator used. Although some trials demonstrated greater reductions in pain and disability with BFRT, others found no significant additional benefit for pain, grip strength, or longer-term function compared with conventional physical therapy or resistance exercise without BFR.^[16,19,22,23]^ This pattern is consistent with previous reviews, which identified potential benefits of upper-extremity BFRT but emphasized the small, heterogeneous evidence base and variability in treatment protocols.^[11–15]^ Collectively, these findings suggest that BFRT may provide additional benefit for selected outcomes, but current evidence does not support its consistent superiority across patients or rehabilitation settings.

Controlled distal-radius-fracture evidence was favorable for selected early outcomes, although samples were small. Cancio et al reported greater reduction in activity pain and PRWE after nonoperative fracture rehabilitation,^[29]^ Sgromolo et al reported larger interval changes in activity pain and PRWE in a 9-participant postoperative trial,^[30]^ and Fan et al reported lower early pain, greater wrist flexion/extension strength, and higher 12-week Cooney function after operative fracture rehabilitation.^[17]^ The lack of a consistent significant between-group difference in passive ROM argues against interpreting these findings as evidence that every aspect of recovery is accelerated. Similarly, Kara et al showed selective gains in biceps thickness and internal-rotation strength without superior pain or SPADI.^[18]^ In hand osteoarthritis, Magni et al reported a higher treatment-response rate and greater pain reduction with BFRT than with advice alone, but did not demonstrate clinically important grip-strength or functional gains.^[33]^ These outcome-specific patterns are more informative clinically than a global statement that BFRT is uniformly effective.

The uncontrolled evidence broadens the range of possible applications but should be weighted much less heavily than comparative trials. Large pre–post changes in LET, shoulder, postoperative elbow, distal-biceps, and rotator-cuff-repair reports occurred alongside conventional rehabilitation, natural healing, progressive loading, education, manual therapy, or other co-interventions.^[20,21,28,31,32,34–38]^ These reports are useful for hypothesis generation, feasibility, prescription detail, and safety description, but the observed recovery cannot be assigned causally to BFRT. This distinction is especially important where dramatic percentage changes are reported from a single patient.

BFRT prescription varied substantially across the included studies.^[16–23,28–39]^ Some studies individualized pressure to approximately 40% to 50% of arterial or LOP, whereas others used fixed pressures, pressure relative to systolic blood pressure, or less standardized approaches.^[16–23,28–39]^ Exercise intensity generally fell in the low-load range, commonly around 20% to 40% 1RM/maximal voluntary contraction, but progression rules differed.^[16–23,28–39]^ The 30-15-15-15 scheme was common, yet frequency ranged from a single experimental session to 5 sessions per week, and treatment duration ranged from 1 session to several weeks.^[16–23,28–39]^ Co-interventions also varied across studies.^[16–23,28–39]^ Because pressure, load, repetition structure, frequency, duration, progression, and co-interventions changed simultaneously across studies, the current evidence cannot isolate an optimal BFRT dose or identify which prescription element explains a favorable outcome.

The contribution of this review is an updated clinical synthesis rather than a claim that upper-extremity BFRT had not been reviewed previously. Ponce-Fuentes et al searched through May 2023 and concluded that the evidence was limited and incompletely reported.^[11]^ Johnson et al subsequently summarized randomized orthopedic upper-extremity trials available through July 2024,^[12]^ Jing et al focused mainly on strength and hypertrophy across broader populations,^[13]^ Sinnott et al mapped the field in a scoping review,^[14]^ and Chen et al meta-analyzed randomized upper-extremity musculoskeletal-disorder trials searched through March 2025.^[15]^ Using the prespecified cutoff before 18 February 18, 2026, the present review adds more recent primary evidence involving symptomatic and postoperative upper-limb musculoskeletal conditions^[19–23]^ and emphasizes actual clinical magnitudes, *P* values, both positive and negative comparative findings, BFRT prescription, co-interventions, safety reporting, and the distinction between controlled and uncontrolled evidence.

Proposed physiological mechanisms – including metabolic stress, motor-unit recruitment, cell swelling, and anabolic signaling – remain biologically plausible,^[8–10]^ but the included clinical studies generally did not directly measure these pathways. Mechanistic explanations are therefore secondary context rather than evidence that a particular mechanism caused the observed clinical improvements. Similarly, the absence of major reported BFRT-related adverse events in several small studies should not be interpreted as proof of safety. Broader musculoskeletal safety reviews and risk-stratification literature support careful patient selection and individualized prescription,^[40,41]^ but upper-limb clinical trials need standardized adverse-event definitions and prospective surveillance.

This review has important limitations. Although 20 reports were included, 10 (50%) were case reports or case series, limiting precision, causal inference, and generalizability. Diagnoses, comparators, follow-up intervals, outcome instruments, BFRT prescriptions, and co-interventions were heterogeneous. The review was limited to English-language published reports. Platform-specific line-by-line historical search logs were not retained for every database, and 15 of the 20 included reports were identified during completion and cross-checking of the prespecified reference-list/citation process rather than the retained database stream; these features limit reproducibility and indicate that the original electronic search had limited sensitivity. Use of the JBI case-report checklist instead of the CARE guideline named in the registered record was a revision-stage protocol deviation and is disclosed in the Section 2. Adverse-event ascertainment and demographic reporting were inconsistent, and heterogeneous age-reporting formats precluded calculation of a valid entire-sample pooled mean ± SD age without unsupported imputation. Where methodological reporting was insufficient for a definite RoB 2 or JBI judgment, the appraisal was recorded conservatively rather than inferred.

Future trials should be adequately powered, diagnosis-specific, and designed to distinguish BFRT from a credible active loading comparator. Reports should provide cuff manufacturer/type and width, method of determining arterial or LOP, prescribed pressure percentage, load and progression, repetition scheme, interset and interexercise rest, cuff inflation/deflation strategy, weekly frequency, total treatment duration, adherence, co-interventions, and prospectively defined adverse events. This level of reporting is necessary to determine whether BFRT adds clinically meaningful benefit and to identify which patients and protocols are most appropriate.

## 6. Conclusion

Current clinical evidence suggests that BFRT may be considered a low-load adjunct in selected adult upper-limb musculoskeletal and postoperative rehabilitation settings. Some randomized trials reported superior benefits of BFRT versus control interventions for selected pain, disability, strength, muscle-morphology, and early postoperative outcomes, whereas other comparative trials found no statistically significant superiority of BFRT for several pain, strength, or functional outcomes. Confidence in the evidence remains limited by small sample sizes, heterogeneous BFRT prescriptions, variable co-interventions, uncontrolled study designs, incomplete adverse-event ascertainment, and limitations in the reproducibility of the historical database search. BFRT should therefore be regarded as a potentially useful rehabilitation and loading strategy rather than a universally superior approach. Larger diagnosis-specific randomized trials using standardized BFRT prescriptions, credible active comparators, and prospective adverse-event monitoring are needed to determine which patients, outcomes, and treatment protocols are most likely to benefit.

## Author contributions

**Conceptualization:** Lama Saif Khan, Abdulelah K. Alqawlaq, Yousef I. Alhusaini, Adnan Alaa Sultan, Ramy Samargandi.

**Data curation:** Abdulelah K. Alqawlaq, Ramy Samargandi.

**Investigation:** Lama Saif Khan.

**Methodology:** Lama Saif Khan, Abdulelah K. Alqawlaq, Ramy Samargandi.

**Project administration:** Lama Saif Khan, Abdulelah K. Alqawlaq, Ramy Samargandi.

**Supervision:** Lama Saif Khan, Fouad Turki Alnajjar, Abdullah Mohammed Falqi, Wahib Shehab Alshaibani, Mohamed Ibrahim Hassan.

**Validation:** Ramy Samargandi.

**Visualization:** Lama Saif Khan, Abdulelah K. Alqawlaq, Ramy Samargandi, Maan Jamjoom.

**Writing – original draft:** Lama Saif Khan, Abdulelah K. Alqawlaq, Majd Hussain Almech, Abdulaziz Khalid Alaryani, Yousef I. Alhusaini, Adnan Alaa Sultan, Ramy Samargandi, Maan Jamjoom.

**Writing – review & editing:** Lama Saif Khan, Abdulelah K. Alqawlaq, Majd Hussain Almech, Adnan Alaa Sultan, Fouad Turki Alnajjar, Abdullah Mohammed Falqi, Wahib Shehab Alshaibani, Mohamed Ibrahim Hassan, Ramy Samargandi, Maan Jamjoom.
